# Camrelizumab-Related Lethal Arrhythmias and Myasthenic Crisis in a Patient with Metastatic Thymoma

**DOI:** 10.1155/2022/4042909

**Published:** 2022-08-05

**Authors:** Bo Zhang, Laxman Gyawali, Zengzhang Liu, Huaan Du, Yuehui Yin

**Affiliations:** Department of Cardiology, The Second Affiliated Hospital of Chongqing Medical University, No. 288 Tian Wen Avenue, Nan'an District, Chongqing 401336, China

## Abstract

Immune checkpoint inhibitors (ICIs) have emerged in recent years as promising treatment options for several malignant tumors. However, ICI therapy has also been associated with various immune-related adverse events (irAEs), especially for patients with preexisting autoimmune status, which sometimes can be life-threatening. A 68-year-old woman diagnosed with metastatic thymoma was treated with camrelizumab, a new ICI, as her antitumor protocol. Eleven days after the first dose of camrelizumab, the patient was admitted to our hospital with symptoms of dyspnea, fatigue, and poor appetite. Workups on admission indicated dramatically elevated transaminase, troponin I, creatine kinase, and a new-onset conduction abnormality on electrocardiography. After detailed evaluation, ICI-related myocarditis, myositis, and hepatitis were diagnosed, and therapies including intravenous methylprednisolone were administered. Coronary angiography was performed to exclude acute coronary syndrome due to dynamic electrocardiography changes on day 3. She lapsed into a coma with respiratory muscle failure on the next day, which was highly suspected of myasthenic crisis. Mechanical ventilation and higher dose of methylprednisolone plus intravenous immunoglobulin were applied immediately. However, the third atrioventricular block occurred within the same day, and an urgent temporary pacemaker was placed. More seriously, refractory ventricular tachycardia (VT) occurred subsequently, and even multiple antiarrhythmic drugs used in combination failed to alleviate the VT storm. On day 5 of hospitalization, she suffered from ventricular fibrillation and died of cardiac arrest. In clinical practice, close follow-up should be conducted after ICI treatment, especially for patients already with or at high risk for autoimmune disorders. A multidisciplinary team approach is of importance for better management of patients with multiple organ involvement.

## 1. Introduction

Immune checkpoint inhibitors (ICIs) are widely used for treatment of diverse types of malignances. ICIs block the interaction between cytotoxic T lymphocyte-associated antigen-4 (CTLA-4), programmed cell death protein 1 (PD-1), and their cognate ligands and thus generate immune responses to tumor cells. However, immune-related adverse events (irAEs) become an inevitable issue, among which colitis, dermatitis, myositis, hepatitis, and endocrinopathies are most frequent [1]. Cardiac and neuromuscular irAEs are rare but potentially life-threatening [2].

The thymus is an immune organ, which means that thymoma may be accompanied by autoimmune disorders or paraneoplastic syndrome. However, cardiovascular involvement occurs with a low incidence of less than 1% [3]. Theoretically, ICIs working as immunotherapy can trigger irAEs in thymoma patients. Here, we report a thymoma patient with multiple organ damage after the first dose of camrelizumab, a newly approved ICI [4].

## 2. Case Presentation

**Figure 1:**
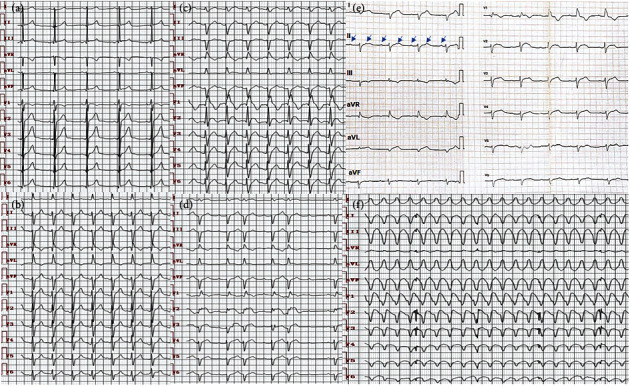
Progressive alterations on ECG. (a) Sinus rhythm and normal cardiac conduction before immune checkpoint inhibitor use. (b) New-onset left anterior fascicular branch block on admission. (c) New-onset complete right branch block plus left anterior fascicular branch block, namely bifascicular branch block. (d) Intermittent second degree atrioventricular block, with frequent premature atrial contractions, ST segment elevation, and poor R-wave progression in precordial leads. (e) Third degree atrioventricular block. Blue arrows indicating regular P waves. (f) Ventricular tachycardia (VT) with heart rate of 230 beats/min.

A 68-year-old woman presented to her local hospital in April 2020 with a 1-week history of increasing exertional dyspnea. Chest computed tomography (CT) revealed an anterior mediastinal mass (44 × 51 mm) with pleural invasion and a large volume of left pleural effusion fluid. The patient was diagnosed with type B2 thymoma (T4N0M1 stage IV) with pleural and left paranephros metastases detected by chest and abdominal enhanced CT, as well as pleural fluid cytology and pleural biopsy. Symptoms were significantly improved by left thoracic pleural drainage. However, due to the late diagnosis and metastasis, she lost her best opportunity for surgery. The patient declined chemotherapy and radiotherapy because of concerns about the side effects. One week after diagnosis, the patient was prescribed with camrelizumab at a dose of 200 mg, with intravenous delivery every 3 weeks. She had no history or positive familial history of cardiovascular disease, neurological disorder, or any other chronic diseases. Indicators including blood cell count, transaminase, creatinine, troponin I (TnI), creatine kinase (CK), creatine kinase MB (CK-MB), thyroid function, electrocardiography (ECG, Figure 1(a)), and echocardiography were unremarkable before initiation of camrelizumab treatment.

However, the patient was admitted to our hospital with complaints of dyspnea, fatigue, muscle weakness, palpitation, and poor appetite 11 days following camrelizumab therapy. Vital signs on admission were stable except for increased respiratory rate of 26 breaths/min and heart rate of 120 beats/min. Manual muscle test indicated slight weakness of the arm and leg skeletal muscles. No other positive signs were detected. Laboratory studies indicated TnI 13.94 ng/mL (normal < 0.030), CK-MB 213.70 ng/mL (normal < 6.30), and CK 8811.40 U/L (normal < 174.0). Alanine aminotransferase (ALT) and aspartate aminotransferase (AST) reached 290 U/L (normal < 40) and 805 U/L (normal < 35), respectively. Serum inflammatory markers, including white blood cell (WBC) count, C-reactive protein (CRP), erythrocyte sedimentation rate (ESR), and procalcitonin (PCT), were all elevated by varying degrees. The thyroid function was normal. Arterial blood gas analysis indicated normal partial pressure of carbon dioxide and decreased oxygen partial pressure of 76 mmHg. Plasma D-dimer and N-terminal pro-brain natriuretic peptide (NT-proBNP) increased, with limited clinical significance, to 690.2 ng/mL (normal < 550.0) and 1169.5 pg/mL (normal < 900.0), respectively (Table 1). Detections for pathogen infections and autoimmune disorders were both negative (Table 2). Transthoracic echocardiography (TTE) revealed preserved left ventricular ejection fraction (LVEF) of 65% with no abnormality of chamber size and ventricular wall motion. ECG on admission showed a new-onset left anterior fascicular branch block (Figure 1(b)), followed by bifascicular branch block on the next day (Figure 1(c)). Cardiac magnetic resonance imaging (MRI) failed to be successfully achieved due to substandard heart rate and breathing rate controlling. In view of the absence of underlying cardiovascular diseases, chemotherapy, radiotherapy, statin use, evidence of virus or other pathogen infections, and a history of ICI administration a few days prior to hospitalization, as well as multiple organ involvement simultaneously, ICI-related myocarditis, myositis, and hepatitis were diagnosed. Intravenous methylprednisolone (80 mg/day), polyene phosphatidylcholine (465 mg/day), reduced glutathione (1.8 g/day), and supportive therapies (including glucose, potassium, and magnesium supplements) were prescribed. In addition, empirical treatment with intravenous antibiotic (piperacillin/tazobactam 4.5 g three times daily) was given to the patient, as concomitant bacterial infection could not be ruled out completely in that stage because of increased inflammatory markers. The patient declined to undergo biopsy of the associated organs due to the potential complications.

**Figure 2:**
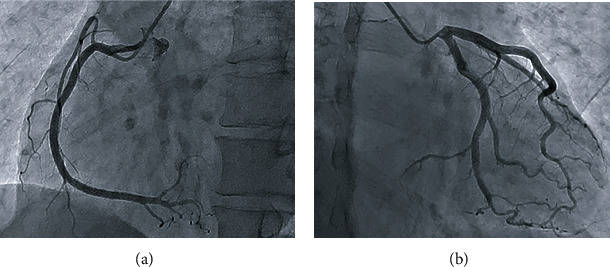
Coronary angiography showing a normal coronary artery anatomy.

On day 3 of hospitalization, ECG revealed an intermittent second-degree atrioventricular block, with frequent premature atrial contractions, ST segment elevation, and poor R-wave progression in precordial leads (Figure 1(d)). Considering the elevated biomarkers of myocardial damage and dynamic ECG alterations, coronary angiography was performed to exclude myocardial infarction even without typical clinical presentations, which revealed normal coronary artery anatomy (Figure 2). Unfortunately, the patient lapsed into a coma with extremely weak respiratory movement on the next day. Arterial blood gas analysis showed fatal carbon dioxide retention with partial pressure of 104 mmHg, with a decreased oxygen partial pressure of 48 mmHg in parallel. Myasthenic crisis was clinically diagnosed for the underlying disease of thymoma and a rapid progression to life-threatening respiratory failure due to respiratory muscle involvement. Tracheal intubation and mechanical ventilation were applied immediately, by which hypercapnia and hypoxia were corrected promptly and consciousness was regained. A higher dose of methylprednisolone (1 g/day) was administered, and intravenous immunoglobulin (IVIG, 20 g/day) was initiated after neurological consultation. Third-degree atrioventricular block occurred abruptly within the same day (Figure 1(e)) even with stable gas exchange and serum electrolyte levels, and then, an urgent temporary pacemaker was inserted. Although multiple therapeutic measures were administered, clinical status deteriorated after the occurrence of ventricular tachycardia (VT) with heart rate of 230 beats/min (Figure 1(f)). The patient presented with loss of consciousness and emergency electrical cardioversion was performed to terminate the lethal arrhythmia. However, even though intravenous administration of high doses of amiodarone, lidocaine, *β*-blocker, and diazepam were used successively, they failed to relieve the VT storm completely. One day after VT, the patient suffered from ventricular fibrillation followed by cardiac arrest, from which she could not be resuscitated (Table 3). Postmortem examination was suggested but was refused by her family members.

## 3. Discussion

ICIs are increasingly used in clinical practice, which in turn can potentially activate autoactive T cells causing immune attack to multiple organs sequentially or simultaneously.

The prevalence of myocarditis caused by ICIs has been reported previously. Data from Bristol-Myers Squibb corporate safety databases showed a rate of 0.09% after administration of nivolumab or ipilimumab and a higher rate of 0.27% when in combination [5]. A multicenter registry reported a prevalence of 1.14% with a median time of onset of 34 days after starting ICIs [6]. Although ICI-related myocarditis seems to be rare, prognosis can be poor and a mortality rate of 50% was reported by Salem and colleagues [7]. In the present case, the patient suffered from ICI-related myocarditis with normal LVEF, but progressive conduction abnormalities and fatal ventricular arrhythmias, and this situation may be even more rare [7]. Endomyocardial biopsy (EMB) is the gold standard to diagnose myocarditis, but this method is rarely conducted due to its invasive nature and possible complications. Lymphocytic infiltration within the myocardium, mainly positive for CD3 or the macrophage marker CD68, is the common pathological presentation of ICI-related myocarditis. Apart from the myocardium, the sinus and atrioventricular nodes can also be infiltrated with lymphocytes, which are potential targets of the activated T cells, so different arrhythmias and conduction abnormalities may occur as a result [5]. Cardiac MRI, which is of high sensitivity and specificity and is usually considered a surrogate to EMB, plays an important role in the diagnosis of myocarditis with traditional causes. However, the diagnostic value of this noninvasive technique in ICI-related myocarditis seems to be not so high as in other common types of myocarditis [8]. In the present case, neither EMB nor cardiac MRI was performed due to subjective and objective reasons. Even without support of the aforementioned examinations, it was reasonable to be diagnosed as ICI-related myocarditis because of the recent medication history of ICI, typical progression of clinical features, elevated myocardial injury biomarkers, dynamic ECG changes, and the exclusion of other confusing causes, which also met the criteria proposed by International Cardio-Oncology Society for clinical diagnosis of ICI-related myocarditis [9]. Intravenous glucocorticoid was prescribed immediately after diagnosis of ICI-related myocarditis, but it could not stop the progression of cardiac conduction disturbance.

The thymus is a central immune organ that is responsible for T-cell development by positive and negative selection. Thymic epithelial tumors (TETs) include thymic carcinoma and thymoma. Thymoma is more likely accompanied by autoimmune disorders, among which myasthenia gravis (MG) is the most common, with a high rate of 30-44% [3]. Other immunological disorders, such as myositis, myocarditis, colitis, hepatitis, and Graves' disease, can also be involved at relatively lower frequencies. Generally, chemotherapy or chemoradiotherapy is recommended for metastatic thymoma, while unsatisfactory responses are often observed in clinical settings. Studies have shown abundant programmed death ligand 1 (PD-L1) expression using immunohistochemistry in TETs [10, 11], which support the potential clinical efficacy of ICIs in TETs. Case series and phase II trials have further demonstrated that ICIs are effective in advanced TETs and thymoma [12–14]. More importantly, the prevalence of ICI-related myocarditis and other irAEs is markedly higher in patients with TETs than with other types of cancers. In one phase II trial, investigating the use of pembrolizumab in patients with recurrent or relapsed TETs, 9.1%, 6.1%, 12.1%, and 3.0% of the patients developed myocarditis, MG, hepatitis, and thyroiditis, respectively [14]. There is a case report of a fatal immune-related storm in a thymoma patient treated with pembrolizumab, leading to hepatotoxicity accompanied by lymphocytosis, thrombocytopenia, and thyroid dysfunction [15]. Multiple organ damage was also involved in our case. Apart from the rapid progression of cardiac adverse events, myasthenic crisis is another fatal complication. The literature suggests that ICI-related MG can be exemplified by *de novo* presentations and exacerbation of preexisting or subclinical MG, with a high proportion of 72.7% for the former [16]. With no MG manifestation prior to the administration of camrelizumab, ICI-related *de novo* MG may be the most likely diagnosis of our patient, although exacerbation of subclinical MG could not be excluded. Immature and TET-derived thymocytes that have escaped self-tolerance and become autoactive may be the mechanism for the development of MG in patients with TETs [17]. ICI therapy can further activate T lymphocytes and strength the autoimmune activity of the cells, given the fact that TET patients are more susceptible to developing autoimmune disorders, which makes thymoma patients more likely than patients with other cancers to experience multiple irAEs [3, 14]. Even a higher dose of glucocorticoid and IVIG were administered immediately after diagnosis of myasthenic crisis, and they could not reverse the outcome due to the extremely worsening situation.

To the best of our knowledge, this is the first report of a thymoma patient developing ICI-related myocarditis, concomitant with MG, myositis, and hepatitis, after the first dose of camrelizumab. Some attention should be paid by clinicians to the following: (1) Complete evaluation must be performed before initiation of ICI, and patients already with or at high risk for autoimmune disorders may not be suitable candidates. (2) Sometimes irAEs can be nonspecific or even asymptomatic, so close regular follow-up with ECG, echocardiography, and routine laboratory tests should be conducted within the first week after ICI treatment, especially for new drugs we are not familiar with. (3) Since irAEs are often characterized by multiple organ involvement, a multidisciplinary approach is important for better management of that group of patients. The manuscript has been previously posted on a preprint server as a preprint [18].

## Figures and Tables

**Table 1:** Results of routine laboratory test.

Parameter	Normal range	Before ICI use	On admission	Day 2	Day 3	Day 4	Day 5
TnI (ng/mL)	<0.030	0.01	13.94	36.05	63.0	46.0	20.8
CK-MB (ng/mL)	0.60-6.30	1.10	213.70	233.6	135.1	71.2	33.8
CK (U/L)	38.0-174.0	20.4	8811.4	4051.3	2245.9	1259.5	867.9
ALT (U/L)	7-40	12	290		273		183
AST (U/L)	13-35	18	805		566		155
Serum K (mmol/L)	3.5-5.3	3.65	4.19	3.74	4.58	4.87	4.92
Serum Mg (mmol/L)	0.75-1.02	0.82	0.85	0.85	0.99	1.01	1.04
Scr (*μ*mol/L)	41.0-81.0	58.4	73.4	43.2	96.2	96.2	105.6
GFR (mL/min)	75.0-300.0	94.1	72.2	141.2	52.9	60.5	47.5
Arterial pH	7.37-7.45		7.46	7.40	7.39	7.01	7.37
PaO_2_ (mmHg)	83-108		76	78	81	48	64
PaCO_2_ (mmHg)	35-48		37	40	38	104	36
BE (mmol/L)	-3.0-3.0		2.3	1.8	1.3	-7.3	-4.1
D-dimer (ng/mL)	<550.0	586.2	690.2	780.1		890.5	900.4
NT-proBNP (pg/mL)	<900.0	308.8	1169.5	1208.2		2230.4	2508.5
WBC (×10^9^/L)	3.50-9.50	7.52	18.8	19.3	21.4	22.6	20.5
Neu (%)	45.0-75.0	71.8	88.2	89.8	89.6	90.1	89.8
RBC (×10^12^/L)	3.80-5.10	4.0	3.98	3.91	3.82	3.56	3.81
Platelet (×10^9^/L)	100-300	149	100	105	98	91	89
CRP (mg/L)	<10	<5.0	15.03	20.0	23.26	24.00	26.80
ESR (mm/h)	<20	12	30	38			57
PCT (ng/mL)	<0.0500	0.0100	0.18		0.20		0.04
Free T3 (pmol/L)	3.1-6.8	3.8	3.3				
Free T4 (pmol/L)	9.5-24.5	13.3	16.1				
TSH (*μ*IU/mL)	0.35-5.00	2.56	2.75				

ICI: immune checkpoint inhibitor; TnI: troponin I; CK-MB: creatine kinase MB; CK: creatine kinase; ALT: alanine aminotransferase; AST: aspartate aminotransferase; K: potassium; Mg: magnesium; Scr: serum creatinine; GFR: glomerular filtration rate; PaO_2_: partial pressure of arterial oxygen; PaCO_2_: partial pressure of arterial carbon dioxide; BE: base excess; NT-proBNP: N-terminal pro-brain natriuretic peptide; WBC: white blood cell; Neu: neutrophil; RBC: red blood cell; CRP: C-reactive protein; ESR: erythrocyte sedimentation rate; PCT: procalcitonin; T3: triiodothyronine; T4: hyroxine, TSH: thyroid-stimulating hormone.

**Table 2:** Results of pathogen test and autoantibody detection.

Category	Result	Category	Result
CVB3-IgM	Neg	Anti-dsDNA	Neg
PVB19-IgM	Neg	Anti-Sm	Neg
ADV-IgM	Neg	Anti-SSA	Neg
HSV-IgM	Neg	Anti-SSB	Neg
Influenza A-IgM	Neg	Anti-Scl-70	Neg
COVID-19 test	Neg	Anti-PM-Scl	Neg
RV-IgM	Neg	Anti-Ro-52	Neg
RSV-IgM	Neg	Anti-mi-2a	Neg
*Chlamydia pneumoniae*-IgM	Neg	Anti-PL-7	Neg
*Mycoplasma pneumoniae*-IgM	Neg	Anti-PL-12	Neg
*Toxoplasma gondii*-IgM	Neg	Anti-SRP	Neg
Blood culture	Neg		
Sputum culture	Neg		
Stool culture	Neg		

CVB3: coxsackievirus B3; PVB19: parvovirus B19; ADV: adenovirus; HSV: herpes simplex virus; COVID: coronavirus disease; RV: rubella virus; RSV: respiratory syncytial virus; Neg: negative.

**Table 3:** Timeline.

18 days prior to admission	Patient diagnosed with type B2 thymoma with pleural and left paranephros metastases at her local hospital, treated with thoracic pleural drainage
11 days prior to admission	First dose of intravenous camrelizumab administered
On admission	Symptoms of dyspnea, fatigue, and poor appetite New-onset left anterior fascicular branch block Elevated CK, CK-MB, TnI, ALT, and AST
Day 2 of hospitalization	Bifascicular branch block Diagnosed with ICI-related myocarditis, myositis, and hepatitis Intravenous methylprednisolone (80 mg/day) Intravenous piperacillin/tazobactam (4.5 g three times daily) Intravenous polyene phosphatidylcholine (465 mg/day) Intravenous reduced glutathione (1.8 g/day) Intravenous glucose, potassium, and magnesium supplements
Day 3 of hospitalization	Intermittent second-degree atrioventricular block and ST segment elevation and poor R-wave progression in precordial leads Coronary angiography confirming normal anatomy of coronary artery
Day 4 of hospitalization	Coma due to respiratory muscle paralysis Mechanical ventilation and higher dose of methylprednisolone (1 g/day) plus IVIG (20 g/day) applied Third degree atrioventricular block Urgent temporary pacemaker placed Ventricular tachycardia Direct current defibrillation and multiple antiarrhythmic drugs used
Day 5 of hospitalization	Ventricular fibrillation followed by cardiac arrest, and finally died

CK: creatine kinase; CK-MB: creatine kinase MB; TnI: troponin I; ALT: alanine aminotransferase; AST: aspartate aminotransferase; ICI: immune checkpoint inhibitor; IVIG: intravenous immunoglobulin.

## Data Availability

Data of the current publication are available from the corresponding author upon reasonable request.

## Supplementary Materials

Supplementary Materials CARE Checklist of information to include when writing a case report.
